# IGF2BP2 Regulates MALAT1 by Serving as an N6-Methyladenosine Reader to Promote NSCLC Proliferation

**DOI:** 10.3389/fmolb.2021.780089

**Published:** 2022-01-17

**Authors:** Le Han, Guangyan Lei, Zhenghong Chen, Yili Zhang, Chen Huang, Wenjuan Chen

**Affiliations:** ^1^ Department of Thoracic Surgery, Tumor Hospital of Shaanxi Province, Affiliated to the Medical College of Xi’an Jiaotong University, Xi’an, China; ^2^ Department of Cell Biology and Genetics/Key Laboratory of Environment and Genes Related to Diseases, School of Basic Medical Sciences, Xi’an Jiaotong University Health Science Center, Xi’an, China; ^3^ Department of Integrated Chinese and Western Medicine, Tumor Hospital of Shaanxi Province, Affiliated to the Medical College of Xi’an Jiaotong University, Xi’an, China; ^4^ Tumor Hospital of Shaanxi Province, Affiliated to the Medical College of Xi’an Jiaotong University, Xi’an, China; ^5^ Department of Third Oncology, Tumor Hospital of Shaanxi Province, Affiliated to the Medical College of Xi’an Jiaotong University, Xi’an, China

**Keywords:** NSCLC, IGF2BP2, M6A, MALAT1, ATG12

## Abstract

Insulin-like growth factor 2 (IGF2) mRNA-binding protein 2 (IGF2BP2) is an important posttranscriptional regulatory for stability and m6A modification. Here, we investigated the role of IGF2BP2 in non–small-cell lung cancer (NSCLC) proliferation. TCGA database was used to predict the expression and clinical significance of IGF2BP2 in normal and NSCLC samples. The expression of IGF2BP2 was further validated in NSCLC samples from surgery. Then we performed the functional study in NSCLC cell lines through overexpressing and knocking down IGF2BP2 in NSCLC cell lines *in vitro* and *in vivo*. The mechanism of interaction between IGF2BP2 and lncRNA metastasis associated lung adenocarcinoma transcript 1 (MALAT1) in NSCLC proliferation was determined by RIP assay. We demonstrated that IGF2BP2 is highly expressed in NSCLC and positively associated with poor overall survival (OS) and disease-free survival (DFS). We identified that lncRNA MALAT1 is a target of IGF2BP2 in NSCLC. IGF2BP2 promotes MALAT1 stability in an m6A-dependent mechanism, thus promoting its downstream target autophagy-related (ATG)12 expression and NSCLC proliferation.

## Introduction

Lung cancer is one of the most diagnosed cancers with high morbidity and mortality in most countries (Bray et al., 2018). Non–small-cell lung cancer (NSCLC), including lung adenocarcinoma (LUAD), lung squamous cell carcinoma (LUSC), and large cell carcinoma histologic subtypes, constitutes about 85% of lung cancer. Despite improvement of basic research and treatment methods of NSCLC, the overall survival rate remains relatively poor (Hirsch et al., 2017; Jones and Baldwin, 2018). Therefore, exploring and figuring out detailed molecular mechanisms of NSCLC is necessary for NSCLC management. Insulin-like growth factor 2 (IGF2) mRNA-binding protein 2 (IGF2BP2) is an RNA-binding protein (RBP) with an important posttranscriptional regulatory role for mRNA localization, stability, and translational control (Li et al., 2019; Liu et al., 2019; Hu et al., 2020). Importantly, IGF2BP2 is a distinct m6A reader that targets lots of mRNA transcripts, promoting the stability and storage of target mRNAs in carcinogenesis. For example, IGF2BP2 promotes liver cancer proliferation in an N6-methyladenosine (m6A)-FEN1-dependent manner (Pu et al., 2020). METTL3 facilitates colorectal carcinoma progression in an m6A-IGF2BP2-dependent way (Li et al., 2019). Recent advances unveiled that IGF2BP2 is a major player in NSCLC progression. For instance, circNDUFB2 inhibits NSCLC progression *via* destabilizing IGF2BPs and activating antitumor immunity (Li B. et al., 2021). MiR-485-5p suppresses growth and metastasis in NSCLC by targeting IGF2BP2 (Huang R. Set al., 2018). However, it remains largely unclear how IGF2BP2 can regulate NSCLC progression.

In this study, we demonstrated that IGF2BP2 is highly expressed in NSCLC and positively associated with poor prognosis. We identified that lncRNA metastasis associated lung adenocarcinoma transcript 1 (MALAT1) is a target of IGF2BP2 in NSCLC. IGF2BP2 promotes MALAT1 stability in an m6A-dependent mechanism, thus promoting its downstream target autophagy-related (ATG)12 expression and NSCLC proliferation.

## Materials and Methods

### NSCLC Tissues

A total of 24 paired samples of tumorous and non-tumorous tissues were collected from surgery at Tumor Hospital of Shaanxi Province with the consent of patients. The histological analysis of each sample was confirmed by pathologists in a double-blind manner. All the procedures were approved by the Ethics Committee of the Tumor Hospital of Shaanxi Province Hospital.

### Cell Lines

Human NSCLC cell lines NCI157, A549, H1299, H460, H1703, H1975, and BEAS control cells were purchased from the Cell Bank of the Chinese Academy of Sciences (Shanghai, China). The cells were grown in RPMI-1640 medium with 10% fetal bovine serum (Gibco, United States). All the NSCLC cell lines were authenticated by short tandem repeat (STR) analysis and were tested for Mycoplasma contamination.

### Quantitative Real-Time PCR

Total RNA of NSCLC cells was extracted with TRIzol reagent (Invitrogen, United States). The relative fold expression was calculated using the comparative threshold cycle (2^−ΔΔCt^). The primer is presented as follows: β-actin: Forward (5′-3′): CCC​ACT​CCT​CCA​CCT​TTG​AC, Reverse (5′-3′): CAT​ACC​AGG​AAA​TGA​GCT​TGA​CAA; IGF2BP2: Forward (5′-3′): GTT​GGT​GCC​ATC​ATC​GGA​AAG​G, Reverse (5′-3′): TGG​ATG​GTG​ACA​GGC​TTC​TCT​G; MALTA1: Forward (5′-3′): GAA​TTG​CGT​CAT​TTA​AAG​CCT​AGT​T, Reverse (5′-3′): GTT​TCA​TCC​TAC​CAC​TCC​CAA​TTA​AT.

### Western Blot

Total protein of NSCLC cells was extracted with RIPA reagent (Beyotime Biotechnology, China). An equal amount of total protein lysate (30 μg) was separated by 7.5–12% SDS-PAGE and transferred onto a PVDF membrane, followed by incubation with primary antibody overnight at 4°C. Then the bands were incubated with secondary antibody for 1 h at room temperature (RT). The bands were detected using a Bio-Rad ChemiDoc XRS system. The primary antibodies were presented as follows: anti-IGF2BP2 (1:1,000, abcam, ab124930), anti-ATG12 (1:1,000, abcam, ab109491), and anti–β-actin (1:2,000, abcam, ab8226).

### Cell Proliferation Assay

Cell proliferation was analyzed using CCK8 assay and colony formation assay. CCK8 assay was performed using a Cell Counting Kit-8 (CCK-8, Biotool, China). Cells were seeded in 96-well plates at a density of 3.5 × 10^3^ cells/well. The CCK8 reagent was added to each well at different time points. After incubation for 4 h at 37°C, the absorbance at 450 nm was measured. For colony formation assay, cells were seeded in 6-well plates and cultured for 14 days. Then the cells were fixed and stained with crystal violet. The number of colonies was countered for five representative fields.

### Subcutaneous Tumor Bearing Nude Mice Model

All animal experiments were approved by the Institutional Animal Care and Use Committee of Xi’an Jiaotong University. Mice (male and 6 weeks old) were subcutaneously injected with NSCLC cells (1.0*10^6^ cells/200 μl). The mice were terminated after 4 weeks of induction, and the tumor volume and tumor weight were measured.

### RNA Immunoprecipitation

IGF2BP2 antibody was used to pull down MALTA1. The IGF2BP2 antibody was then recovered with protein A/G beads, and RNA level of MALTA1 in the precipitates was measured by qRT-PCR. For m6A RIP, m6A antibody (MABE1006) (Millipore Sigma, Burlington, MA) was used to pull down m6A modified MALTA1, and RNA level of MALTA1 in the precipitates was measured by qRT-PCR.

### Statistical Analysis

Statistical analyses were performed using the GraphPad Prism program. The *t*-test was used to compare the mean of a continuous variable between two groups. OS and disease-free survival (DFS) curves were calculated using the Kaplan–Meier method and were analyzed with the log-rank test. *p* values < 0.05 were considered as significant.

## Results

### IGF2BP2 Is an Unfavorable Prognostic Marker in NSCLC

**FIGURE 1:**
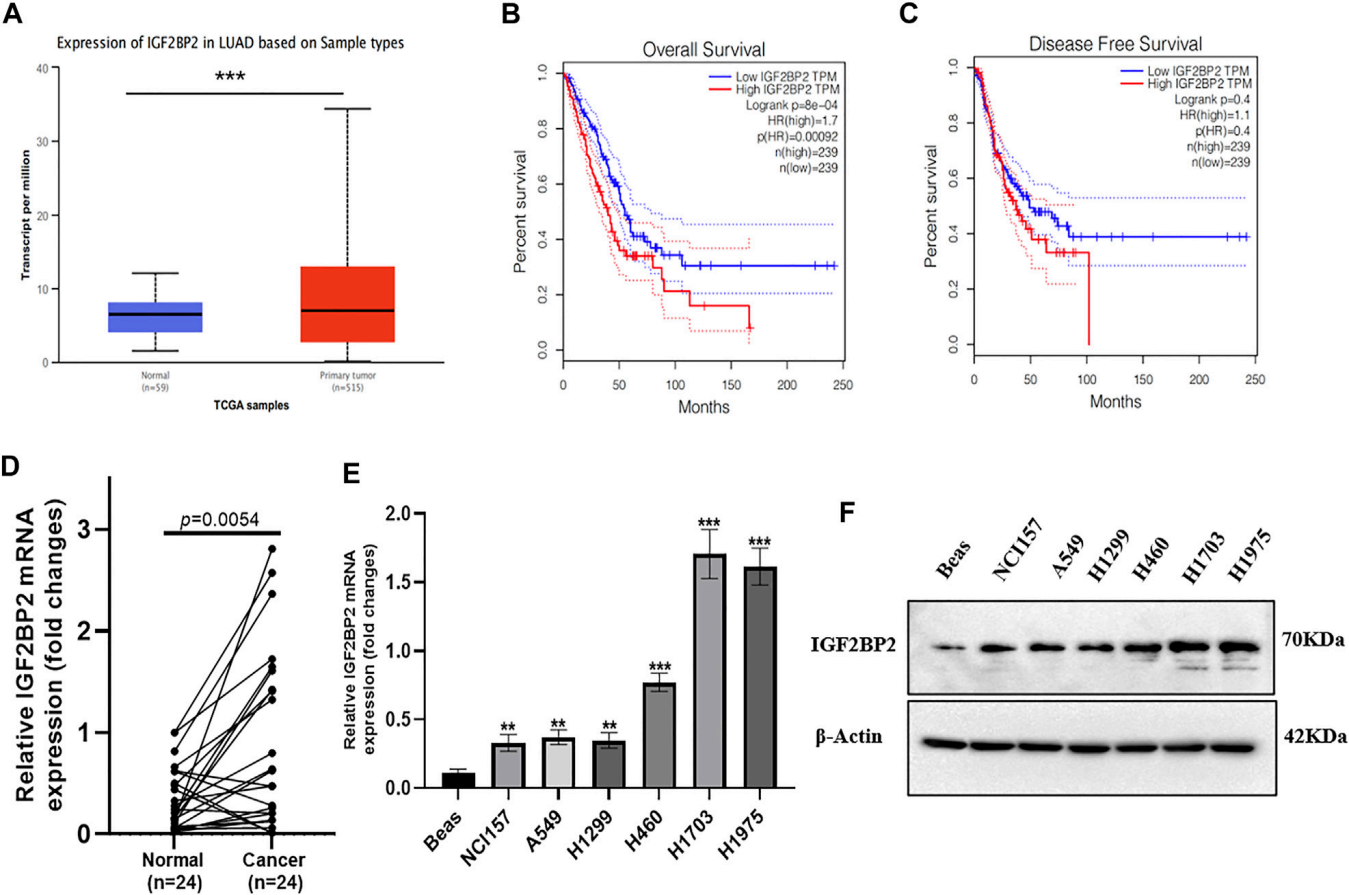
IGF2BP2 is an unfavorable prognostic marker in NSCLC. **(A)** The expression difference of IGF2BP2 in the TCGA database. **(B,C)** The expression of IGF2BP2 is related to the survival and prognosis of patients with non-small cell lung cancer. **(D)** The expression difference of IGF2BP2 in non-small cell lung cancer tissues and adjacent tissues (*n* = 24). **(E,F)**. The expression of IGF2BP2 in non-small cell lung cancer cell lines. Data are presented as mean ± SD. *p* values are calculated by unpaired two-sided *t*-test.

We first checked the TCGA database to investigate the role of IGF2BP2 in NSCLC patients. As compared with normal tissues, the IGF2BP2 mRNA level was upregulated in primary NSCLC tissues (Figure 1A). Moreover, a higher IGF2BP2 mRNA level was positively correlated with poor overall survival (OS) and disease-free survival (DFS) (Figures 1B,C). The upregulation of IGF2BP2 mRNA was further validated in fresh NSCLC samples and the paired adjacent non-tumor sites (Figure 1D, *n* = 24, *p* < 0.01). Consistently, the upregulation of IGF2BP2 was presented in NSCLC cell lines compared with the BEAS cells (Figures 1E,F). These results highlight the clinical significance of IGF2BP2 in NSCLC.

### IGF2BP2 Promotes NSCLC Cell Proliferation *In Vitro* and *In Vivo*

**FIGURE 2:**
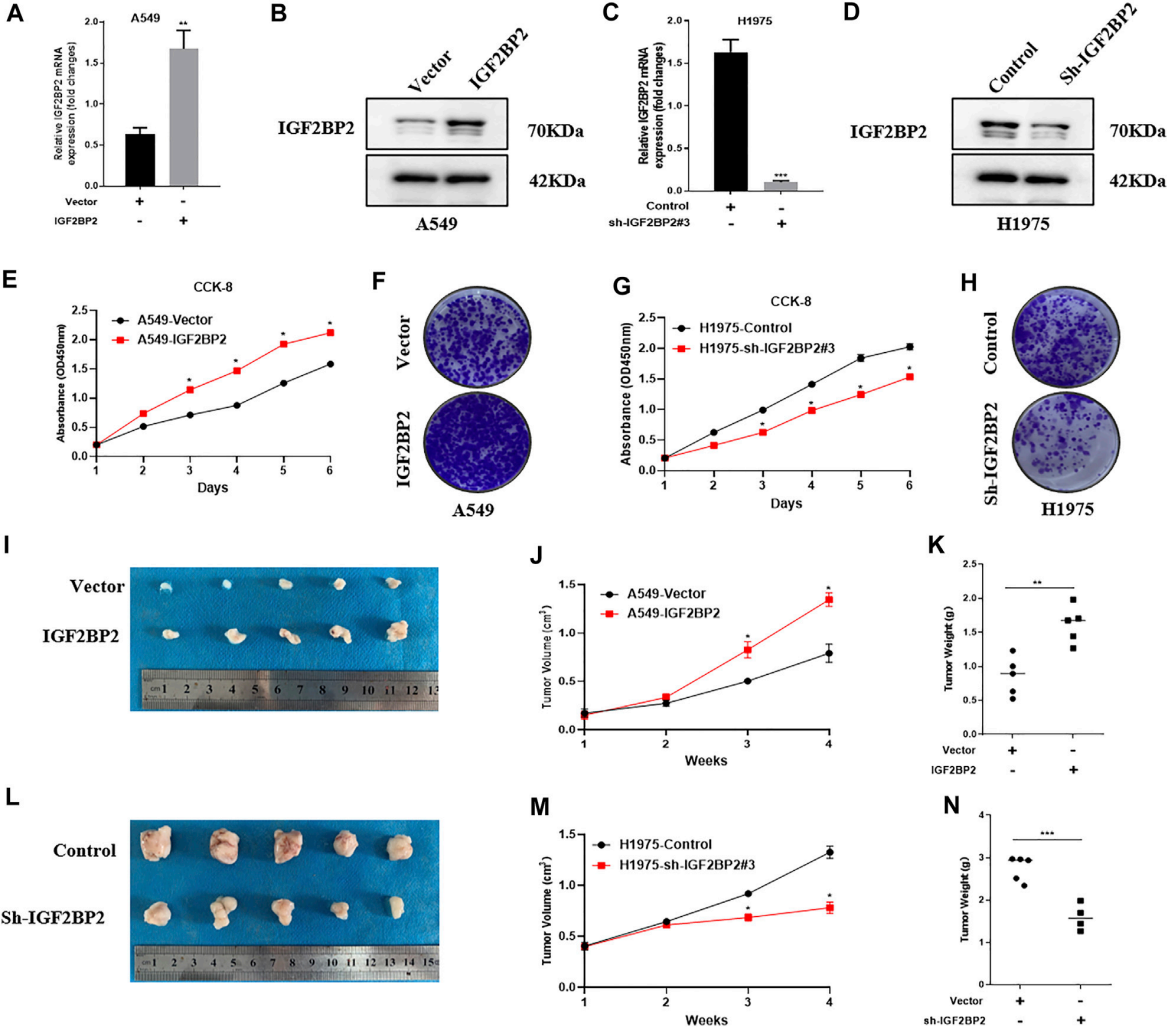
IGF2BP2 promotes NSCLC cell proliferation *in vitro* and *in vivo*. **(A,B)** Real-time quantitative PCR and western blot analysis overexpress IGF2BP2 in A549 cells. **(C,D)**. Real-time quantitative PCR and western blot analysis knocked out IGF2BP2 in H1975 cells. **(E–H)**. CCK-8 and clone formation experiments analyze the effect of IGF2BP2 on the proliferation of non-small cell lung cancer cell lines. **(I–N)**. *In vivo* tumor formation experiments in nude mice analyzed the effect of IGF2BP2 on the proliferation of non-small cell lung cancer cell lines. Data are presented as mean ± SD. *p* values are calculated by unpaired two-sided *t*-test.

To gain an insight into IGF2BP2 in NSCLC progression, we ectopically expressed IGF2BP2 in A549 cells (Figures 2A,B) and generated IGF2BP2 knockdown in H1975 cells (Figures 2C,D). We found that IGF2BP2 promoted cell proliferation, as determined using CCK8 and colony formation assays (Figures 2E,F). However, knockdown of IGF2BP2 showed an opposite effect on NSCLC cell proliferation (Figures 2G,H). Then we established the subcutaneous tumor bearing nude mice model to better understand the oncogenic role of IGF2BP2 in NSCLC. The results confirmed that IGF2BP2 promoted NCCLC proliferation, as indicated by larger tumor volume and heavier tumor weight (Figures 2I–K). Conversely, IGF2BP2 knockdown repressed tumor growth in nude mice (Figures 2L–N). All the results indicate the oncogenic role of IGF2BP2 in NSCLC.

### IGF2BP2 Regulates MALTA1 Expression in NSCLC

**FIGURE 3:**
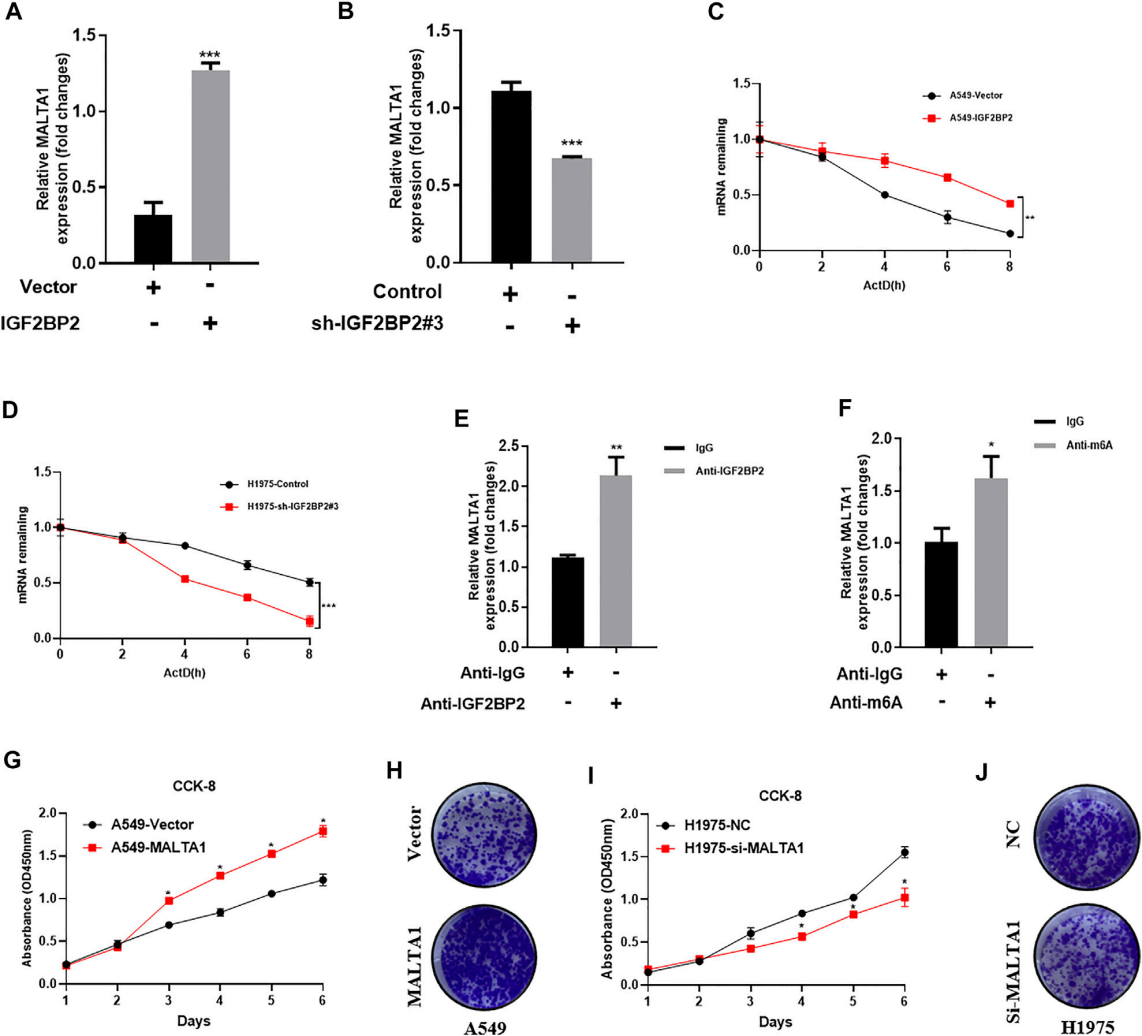
IGF2BP2 regulates MALTA1 expression in NSCLC. **(A,B)** Real-time quantitative PCR analysis IGF2BP2 regulates the expression of MALAT1. **(C,D)** MALAT1 half-life determination. **(E)** RIP-PCR analysis of the interaction between IGF2BP2 and MALAT1. **(F)** mRIP-PCR analysis of m6A modification of MALAT1. **(G–J)** CCK-8 and clone formation experiments analyze the effect of MALAT1 on the proliferation of non-small cell lung cancer cell lines. Data are presented as mean ± SD. *p* values are calculated by unpaired two-sided *t*-test.

It is widely recognized that lncRNA MALAT1 is a key regulator in NSCLC initiation, progression, and metastasis (Tang et al., 2018; Song J. et al., 2020; Li M. et al., 2021). Because IGF2BP2 usually functions as a key regulator of IncRNAs, we speculate whether lncRNA MALAT1 can be regulated by IGF2BP2. As expected, IGF2BP2 overexpression upregulated the MALAT1 level, whereas MALAT1 was significantly downregulated by IGF2BP2 knockdown (Figures 3A,B). We further investigate the mechanisms by which IGF2PB2 regulates MALAT1 expression. Since a central role of IGF2BP2 in carcinogenesis is to regulate RNA stability, we examined the stability of MALAT1 by using actinomycin D (2 μg/ml). The results showed that the MALAT1 decay was slowed down *via* upregulating of IGF2BP2 (Figure 3C). Conversely, knockdown of IGF2BP2 accelerates MALAT1 decay compared with control cells (Figure 3D). In addition, RIP-PCR assay further validated the interaction between IGF2BP2 and MALAT1 (Figure 3E). As for N6-methyladenosine (m6A), it is the most abundant internal modification on RNAs (Jin et al., 2019; Xue et al., 2021). We detected whether IGF2BP2 promotes MALAT1 stability *via* m6A modification. RIP assays with m6A antibody identified enrichment of MALAT1 *via* upregulating of IGF2BP2 (Figure 3F). All these data suggested that IGF2BP2 regulates MALTA1 stability in NSCLC.

### IGF2BP2 Promotes NSCLC Proliferation *via* Upregulating ATG12 Expression

**FIGURE 4:**
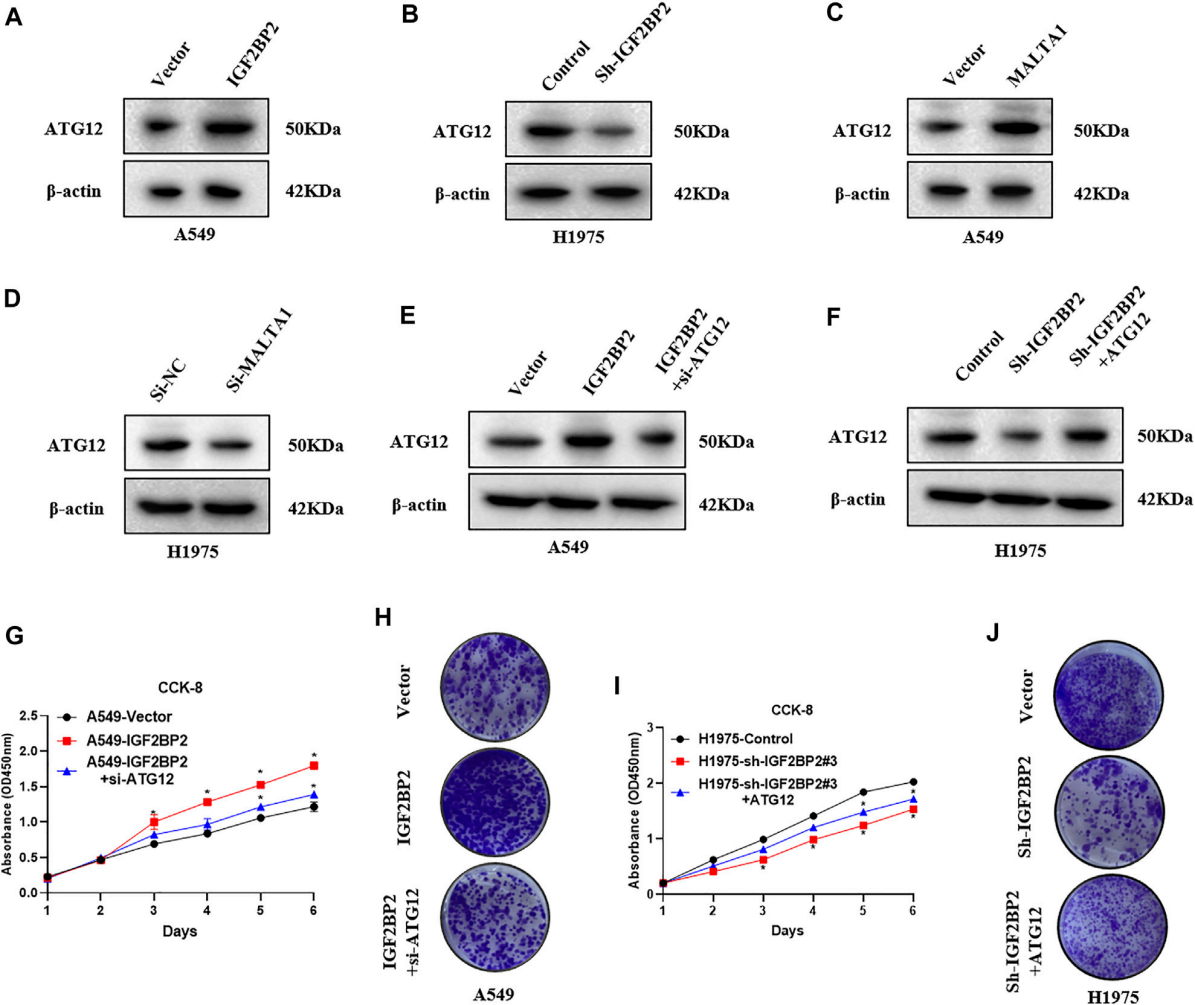
IGF2BP2 promote NSCLC proliferation via upregulating ATG12 expression. **(A,B)** Western blot analysis of the effect of IGF2BP2 on the expression of ATG12 protein. **(C,D)** Western blot analysis of the effect of MALAT1 on the expression of ATG12 protein. **(E,F)** Western blot analysis of the effect of MALAT1 and IGF2BP2 on the expression of ATG12 protein. **(G–J)** CCK-8 and clone formation experiments analyze the effect of MALAT1 and IGF2BP2 on the proliferation of non-small cell lung cancer cell lines. Data are presented as mean ± SD. *p* values are calculated by unpaired two-sided *t*-test.

It is known that ATG12 is a key downstream regulator of MALAT1, and ATG12 is required for NSCLC progression (He et al., 2020). Therefore, we tested whether IGF2BP2 can regulate ATG12 expression *via* MALAT1 in NSCLC. Western blot assay revealed that IGF2BP2 overexpression upregulated the ATG12 level, whereas ATG12 was significantly downregulated by IGF2BP2 knockdown (Figures 4A,B). We also confirmed that MALAT1 overexpression upregulated the ATG2 level, whereas ATG12 was significantly downregulated by MALAT1 knockdown (Figures 4C,D). To further validate whether IGF2BP2 can regulate NSCLC proliferation *via* ATG12, we knocked down ATG12 expression in IGF2BP2 overexpressing cells and ectopically expressed ATG2 in IGF2BP2 knockdown cells (Figures 4E,F). CCK8 assay indicated that knockdown ATG12 expression can repress cell proliferation in IGF2BP2 overexpressing cells, while ectopically expressed ATG12 promotes cell proliferation in IGF2BP2 knockdown cells (Figures 4G,I). Colony formation assay showed the same trend as the CCK8 experiment (Figures 4H,J). All the data suggested that IGF2BP2 promotes NSCLC proliferation *via* the lncRNA MALAT1/ATG12 axis.

## Discussion

In the present study, we showed that high expression of IGF2BP2 in NSCLC is correlated with unsatisfied OS and DFS. lncRNA MALAT1 is a direct target of IGF2BP2 in NSCLC. Mechanistically, IGF2BP2 promotes MALAT1 stability *via* m6A modification and promoting its downstream target ATG12 expression.

Using TCGA database, we identified the clinical significance of upregulating IGF2BP2 in NSCLC. To support the findings of TCGA database, we further highlight elevated IGF2BP2 expression in NSCLC samples and cell lines. To further unveil the function of IGF2BP2 in NSCLC progression, we performed the functional study in NSCLC cell lines through overexpressing and knocking down IGF2BP2 in NSCLC cell lines. We showed that IGF2BP2 promotes cell proliferation and viability *in vitro* and *in vivo*, indicating the oncogenic role of IGF2BP2 in NSCLC. Since IGF2BP2 functions as a key regulator of IncRNAs, we speculate whether lncRNA MALAT1 can be regulated by IGF2BP2. MALAT1 is a most widely studied lncRNA in tumorigenesis. MALAT1 act as a metastasis-suppressing lncRNA in breast cancer (Kim et al., 2018). However, MALAT1 serves as an oncogenic lncRNA in NSCLC proliferation and Gefitinib resistance by acting as a miR-200a sponge (Feng et al., 2019). Consistently, lncRNA MALAT1 also plays a pro-oncogenic role in ovarian cancer (Jin et al., 2017), osteosarcoma (Zhang et al., 2020), acute lymphoblastic leukemia (Song Y. et al., 2020), and colorectal cancer (Guo et al., 2020). Our results showed that IGF2BP2 overexpression increased the MALAT1 level, whereas MALAT1 was significantly downregulated by IGF2BP2 knockdown, suggesting that lncRNA MALAT1 can be regulated by IGF2BP2. Regulating the RNA stability is the major way of IGF2BP2 to interact with target RNAs (Huang H. et al., 2018; Chen et al., 2019). To figure out the mechanisms by which IGF2PB2 regulates MALAT1 expression, we tested the stability of lncRNA MALAT1 by overexpressing and knocking down IGF2BP2 and found that the MALAT1 decay was slowed down *via* upregulating of IGF2BP2. Conversely, knockdown of IGF2BP2 accelerates MALAT1 decay. Moreover, RIP-PCR assay further validated the interaction between IGF2BP2 and MALAT1. As for N6-methyladenosine (m6A), it is the most abundant internal modification on RNAs (Jin et al., 2019; Xue et al., 2021). We detected whether IGF2BP2 promotes MALAT1 stability *via* m6A modification. RIP assays with m6A antibody identified enrichment of MALAT1 *via* upregulating of IGF2BP2. All these data suggested that IGF2BP2 regulates MALTA1 stability in NSCLC. It is known that ATG12 is a key downstream regulator of MALAT1, and ATG12 is required for NSCLC progression (He et al., 2020). Therefore, we tested whether IGF2BP2 can regulate ATG12 expression *via* MALAT1 in NSCLC. The results revealed that IGF2BP2 overexpression upregulated the ATG12 level, whereas ATG12 was significantly downregulated by IGF2BP2 knockdown. In addition, to further validate whether IGF2BP2 can regulate NSCLC proliferation *via* ATG12, we knock down ATG12 expression in IGF2BP2 overexpressing cells and ectopically expressed ATG2 in IGF2BP2 knockdown cells. We showed that knockdown ATG12 expression can repress cell proliferation in IGF2BP2 overexpressing cells, while ectopically expressed ATG12 promotes cell proliferation in IGF2BP2 knockdown cells, suggesting that IGF2BP2 promotes NSCLC proliferation *via* the lncRNA MALAT1/ATG12 axis.

In summary, we found that upregulation of IGF2BP2 in NSCLC is correlated with unsatisfied OS and DFS. IGF2BP2 promotes NSCLC proliferation *via* regulation of lncRNA MALAT1 stability in an m6A-dependent manner. Targeting the IGF2BP2/lncRNA MALAT1/ATG12 axis may be beneficial for NSCLC treatment.

## Data Availability

The original contributions presented in the study are included in the article/Supplementary Material, further inquiries can be directed to the corresponding authors.

## Abbreviations

NSCLC, non–small-cell lung cancer; LUAD, lung adenocarcinoma; LUSC, lung squamous cell carcinoma; IGF2BP2, insulin-like growth factor 2 (IGF2) mRNA-binding protein 2; RBP, RNA-binding protein; OS, overall survival; DFS, disease-free survival; MALAT1, metastasis associated lung adenocarcinoma transcript 1; ATG, autophagy-related; m6A, N6-methyladenosine.
